# DripOMeter: An open-source opto-electronic system for intravenous (IV) infusion monitoring

**DOI:** 10.1016/j.ohx.2022.e00345

**Published:** 2022-08-09

**Authors:** Kavyashree Venkatesh, Sourabh Suresh Alagundagi, Vikas Garg, Krupakar Pasala, Deval Karia, Manish Arora

**Affiliations:** Centre for Product Design and Manufacturing, Indian Institute of Science, Bangalore 560012, India

**Keywords:** Intravenous therapy, IV Drip chamber, Device for medical purpose, Medical alarms

## Abstract

Intravenous (IV) infusion is a common medical procedure which involves the administration of fluids directly into the blood stream typically through a vein in the arm of the patient. Though gravity fed IV-drip is a safe, effective and an affordable tool, yet several complications can arise in its usage and thus requires constant monitoring. In this paper, a solution is presented for infusion monitoring based on detection of drops falling through the drip chamber. The system presented here accurately tracks the fluid flow and assists the users in monitoring the infusion sessions. The system generates alarm upon detecting significant deviation from set drip rate. The system keeps track of total volume infused and alerts when a desired volume is about to be administered. The device offers a solution to reduce the risks associated with the IV infusion therapy especially in low-resource setting and provide peace of mind to caregivers.

## Graphical abstract

**Figure:**
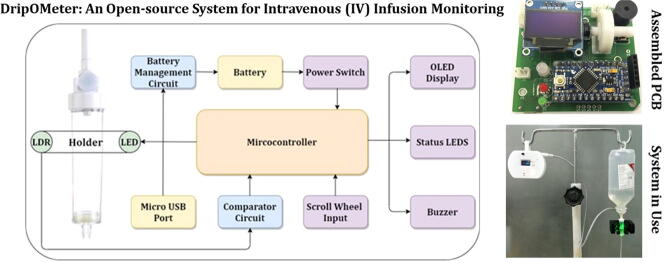

**Specifications table:** 

Hardware name	DripOMeter
Subject area	• Medical devices (Design & Development) • Engineering • Educational tools and open-source alternatives to existing infrastructure
Hardware type	• Medical parameter monitoring • IV Drip monitoring • Field measurements and sensors • Electrical engineering and computer science
Closest commercial analog	DripO from Evelabs [1] Drip Infusion Rate Monitor from Shift Labs [2] Monidrop [3]
Open source license	CERN Open Hardware License Version 2 – Weakly Reciprocal (CERN-OHL-W)
Cost of hardware	$ 59.22
Source file repository	https://doi.org/10.17605/OSF.IO/DYCMS

## Hardware in context

In clinical medicine, one of the principal activity is the treatment of diseases using pharmacology, which is carried out by administering medication by oral, subcutaneous, intramuscular, intra-arterial or intravenous (IV) routes. Intravenous therapy is the infusion of liquid substances directly into a vein and is the fastest way to deliver fluids and medications throughout the body [4]. Intravenous therapy may be used for fluid administration to correct dehydration, to correct electrolyte imbalances, to deliver medications or for blood transfusions. IV therapy is relatively inexpensive and fast procedure which provides 100 % bioavailability of administered substances. Some of the medications can only be administered through IV route.

A standard infusion set used for IV therapy connects pre-filled, sterile reservoir (glass bottle, plastic bottle or plastic bag) of fluids to the body of the patient. It consists of an attachment called drip chamber that allows the fluid to flow one drop at a time, making it easy to see the flow rate and trap air bubbles; a long sterile tube with a clamp to regulate or stop the flow; and a connector to attach to the access device e.g. an IV cannula. Sometimes Y-sets are used to allow “piggybacking” of another infusion set onto the same infusion line or administration of additional medicines directly into same access port.

Despite its popularity, IV therapy also poses a few challenges which get aggravated when operating in low resource setting. These challenges include:

• Air embolism: Air embolism is the entry of air from the environment into the venous system which is caused due to completely empty reservoir, open IV system and other reasons. It can lead to stroke or cardiac arrest
• Drip rate customization: Different fluids to be administered require different drip rate setting. e.g. Drip rate for glucose infusion is different from that of blood infusion.
• Drip rate deviation: The drip rate set at the beginning of the session may vary due to various reasons, e.g. patient movement.
• Fluid overload: Due to the excessive administration of fluids into the circulatory system, fluid overload can occur.
• Reservoir wear-out: The fluid reservoir need be replaced time to time depending on the fluid infused and the rate set.

Due to the drip rate changes, less or more than required fluid might get delivered into the patient termed as under-infusion or over-infusion respectively. Under-infusion can lead to sub-optimal therapy, dehydration or metabolic disturbances. And over-infusion can cause speed shock, electrolyte imbalance, high blood pressure or metabolic disturbances [5]. This creates a need for a system which can closely monitor the fluid administration by IV therapy in clinical as well as home-care settings to prevent undesired consequences.

To overcome the challenges associated with the IV therapy, multiple groups have worked towards the development of the infusion monitoring system using different technologies. Zeev Burko invented a device [6] for volumetric measurement of a drop of fluid administered in a gravity intravenous set using infrared light-light sensor arrangement. Shroff *et al.* developed an IV monitoring system called AccuFlow [7] using optical sensor which was commercialised and evaluated on paediatric patients. Shift-labs developed Drip Assist [2], which also used optical sensing technology and is the FDA cleared infusion rate monitor with 99 % accuracy, without the complexity or cost of a pump. DripO, a wireless infusion monitor was developed by Evelabs, to measure and display the flow rate and share data wirelessly [1]. A drip rate meter was developed by Kamble et al. [8] to work for different types of fluids and drip chamber walls of different optical transmittivity using an LED and a photodiode placed around IV drip chamber. The device displays drip rate and has alarms to alert, once the rate deviates from pre-set value. Vignali et. al. designed an IV stand [9] with an alarm for low fluid level detection using a combination of LED and phototransistor for monitoring the infusion.

Apart from the optical sensing technique, various other technologies have been used by research groups for infusion monitoring. Techniques like Ultrasonic sensing [10], Capacitive sensing [11], Piezo-electric sensing [12], microwave time domain reflectometry [13] are used to solve the IV infusion related challenges. Using CMOS Liquid Level sensor technique, an Intravenous Drip detecting system was developed by Chiang and Tsai [14]. RFID technology was used by Huang and Lin to develop a warning system for running-out of injection fluid] [15].

While the above approaches show the possibility of monitoring infusion therapy, they are either too complex or unaffordable for widespread use. Amidst all this, the optical technique based IV infusion monitoring seems to be promising. In this paper we present an open source design of IV drip monitoring system.

## Hardware description

**Fig. 1:**
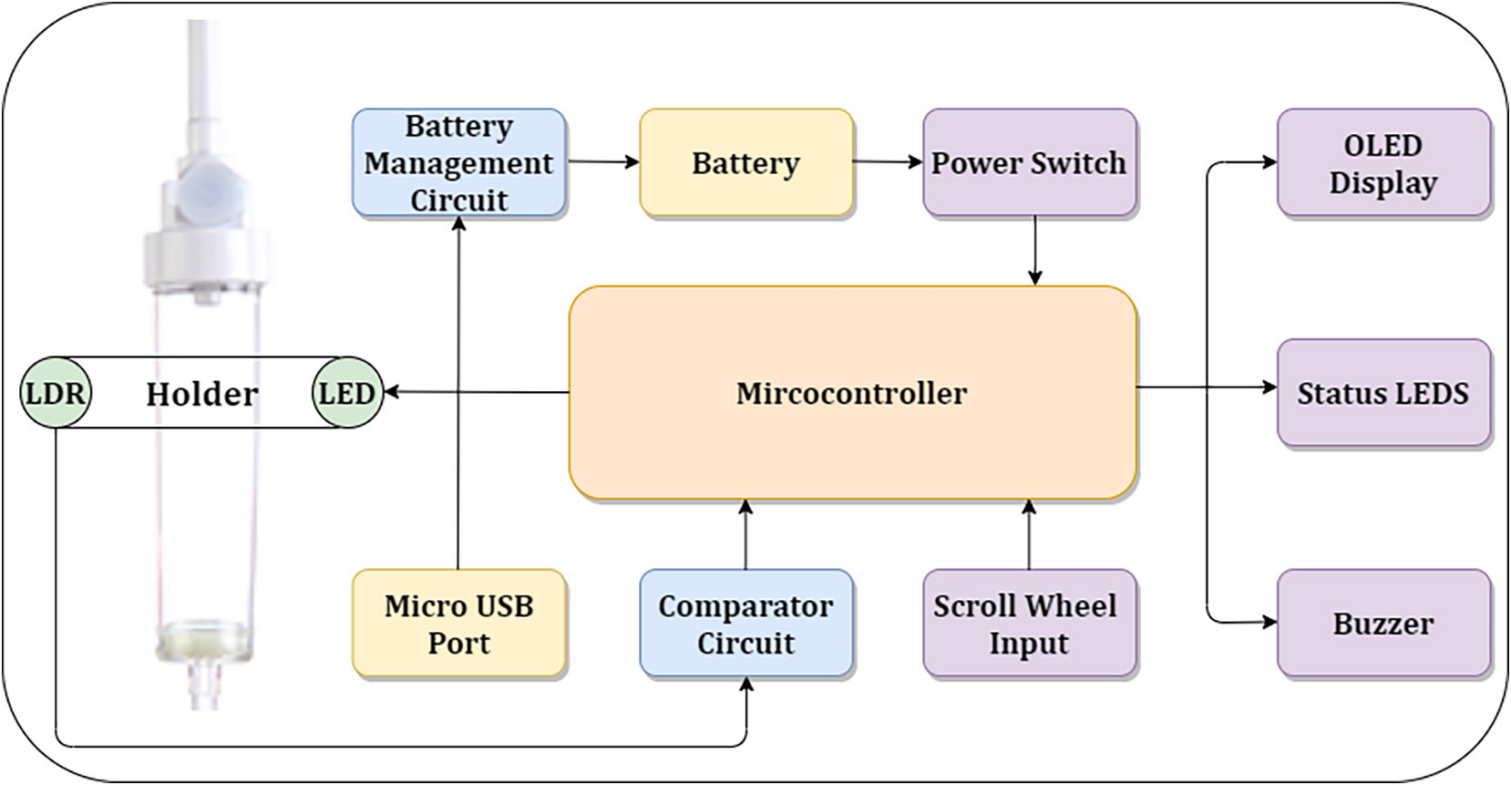
System Architecture of DripOMeter.

The system architecture of the IV drip monitoring system (DripOMeter) is as shown in the Fig. 1. The system is an integration of the following modules:

### Drop detector module

**Fig. 2:**
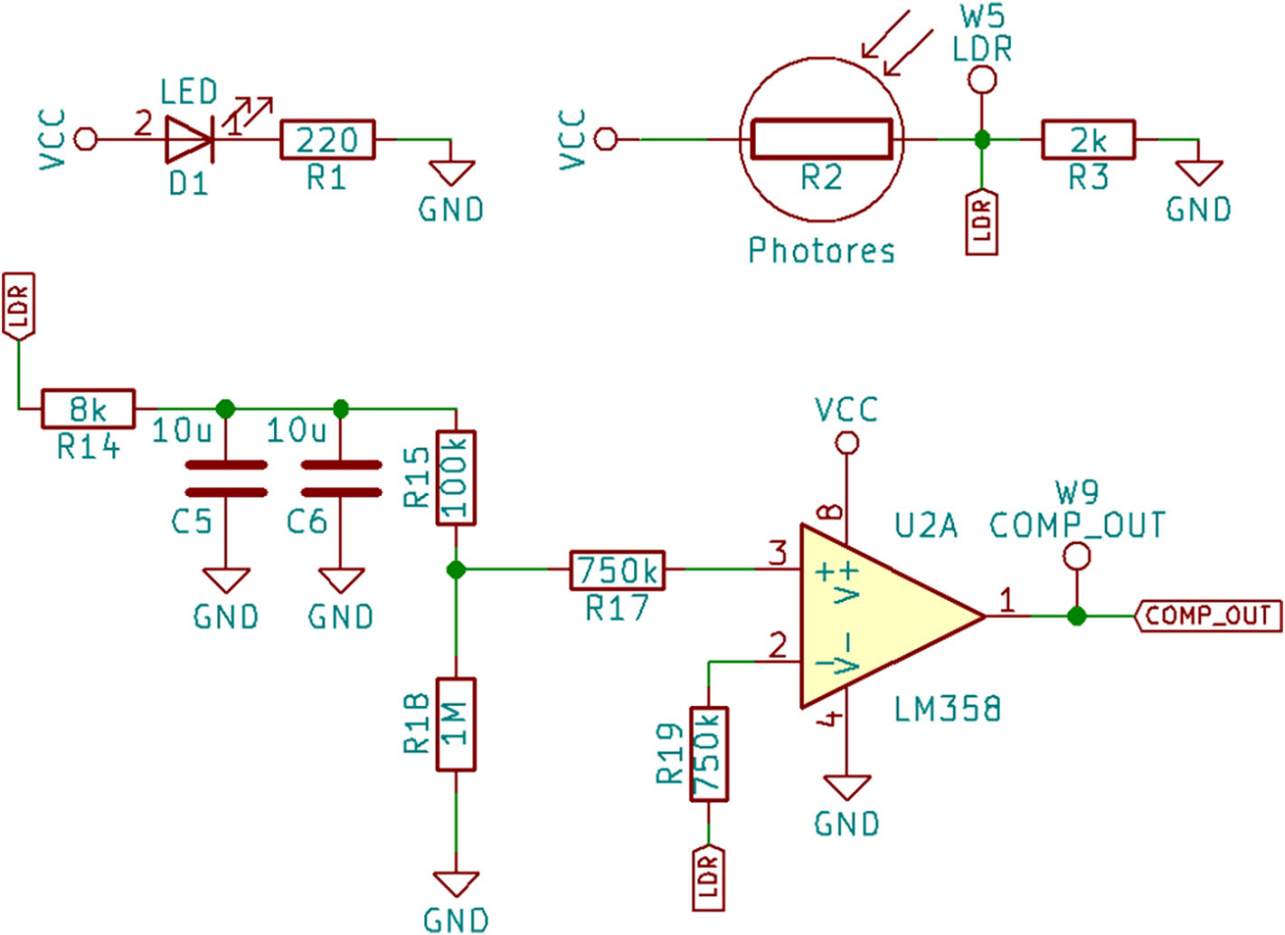
Drop detector & Comparator module.

The drop detector module comprises of a light source, a light detector and a voltage divider circuit. Light source and light detector are mounted linearly across the drip chamber with the help of separate holder. A green coloured visible Light Emitting Diode (LED) is used as light source and Light Dependent Resistor (LDR) is used as light detector. The LED is powered using the power line from the controller module and the LDR along with the standard resistor forms the voltage divider circuit. The circuit is designed, such that the dark resistance of the LDR matches the resistance of the standard resistor connected in series. This design ensures that the voltage signal to the next stage, taken from the junction of LDR and the standard resistor remains in the required range for proper functioning of the Op-Amp as comparator. In absence of the drop, the light beam from the LED is directly incident on the LDR, due to which the resistance of the LDR will be low. Once a drop falls through the drip chamber, the light beam is cut-off and the resistance offered by the LDR increases. Thus, there is a high voltage to low voltage transition, which forms an input for the comparator module (Fig. 2).

### Comparator module

The comparator module consists of the Operational Amplifier LM358 being used as voltage comparator. The signal obtained from the LDR is passed through a low pass filter with cut-off frequency of 1 Hz to provide a dynamic baseline voltage for comparison. This is followed by a voltage divider circuit which feeds only 90 % of the incoming signal to the non-inverting input for the OpAmp. The inverting input for the OpAmp is also the signal from the LDR. In absence of the drop, the comparator output will be a low voltage state. During the passage of the drop, the low pass signal at non-inverting input is not effected whereas signal at inverting input shows a dip in voltage. Thus, when the drop is detected by this module, the comparator output rises from low voltage state to high voltage, thus creating square pulse signal. This square pulse forms the input for the interrupt pin of the micro-controller module meant for the detection of drops.

### Controller module

The 3.3 V, 8 MHz Arduino Pro Mini micro-controller forms the controller module of the system. The micro-controller is the heart of the system which coordinates the various processes and helps achieve the functionalities of the system. The Arduino is programmed in Embedded C using the Arduino IDE. The Arduino code will be explained in the software section. The Arduino Pro Mini is programmed using a USB-UART adapter module.

### User Interface module

The drip monitoring system uses the 3D printed scroll wheel arrangement that forms the user input part of the system.

**Fig. 3:**
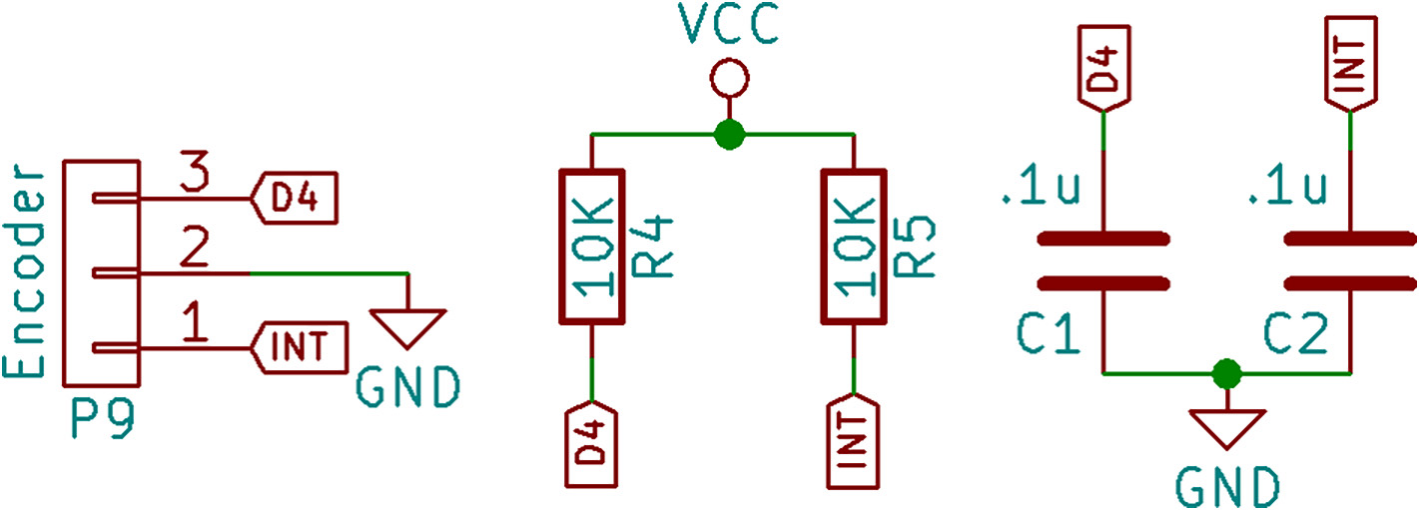
Encoder circuitry.

The scroll wheel arrangement consists of an incremental mechanical rotary encoder, low pass filters and a tactile switch. An incremental mechanical rotary encoder, EC10E1220505 with 24 detents and 12 pulses per rotation is used to capture rotation input form the user. The encoder pins A and B are pulled up high using the pull-up resistors and are connected to an interrupt pin and a digital pin of the microcontroller. The low pass filters remove the high frequency noise in the encoder signal generated by the encoder (Fig. 3). The controller firmware is designed to function properly using only one interrupt input. This encoder circuitry and the switch can be used as a User Interface tool in other applications as well.

This arrangement facilitates the user to scroll through and select from the list of menus displayed on the display. A 0.96″ mono-color, I2C Organic Light Emitting Diode (OLED) display along with the status indicator LEDs, and the buzzer forms the user output part of the UI. The Red-Green bicolor common cathode 5 mm LED is used as status indicator, with red LED indicating low battery status and green LED indicating the drop being detected during the monitoring session. Apart from this, two small 3 mm LEDs indicate the battery charge status. The Piezo-buzzer used acts as the alarm for the session alerts.

### Battery management module

The battery module consists of the battery, battery management circuit, MicroUSB, power switch and the voltage divider. Rechargeable Lithium-ion battery (3.7 V, 2600 mAh) is used for providing power over long sessions. The battery management circuit consists of a fully integrated, lithium-ion charge management IC, MCP73831 which has a charge rating of 4.2 V, 500 mA. The IC has end-of-charge control and automatically shuts down the charging process, once the battery is charged to 4.2 V. Also, it provides the charge status information by controlling the small LEDs connected to its status pin. To charge the battery, a 5 V DC adapter with an AB type MicroUSB connector is used. A slide switch is used as the power switch in the system. Also, to read the voltage form the battery terminal, a voltage divider circuit has been used for making it compatible with the microcontroller pin.

### Assembled hardware

**Fig. 4:**
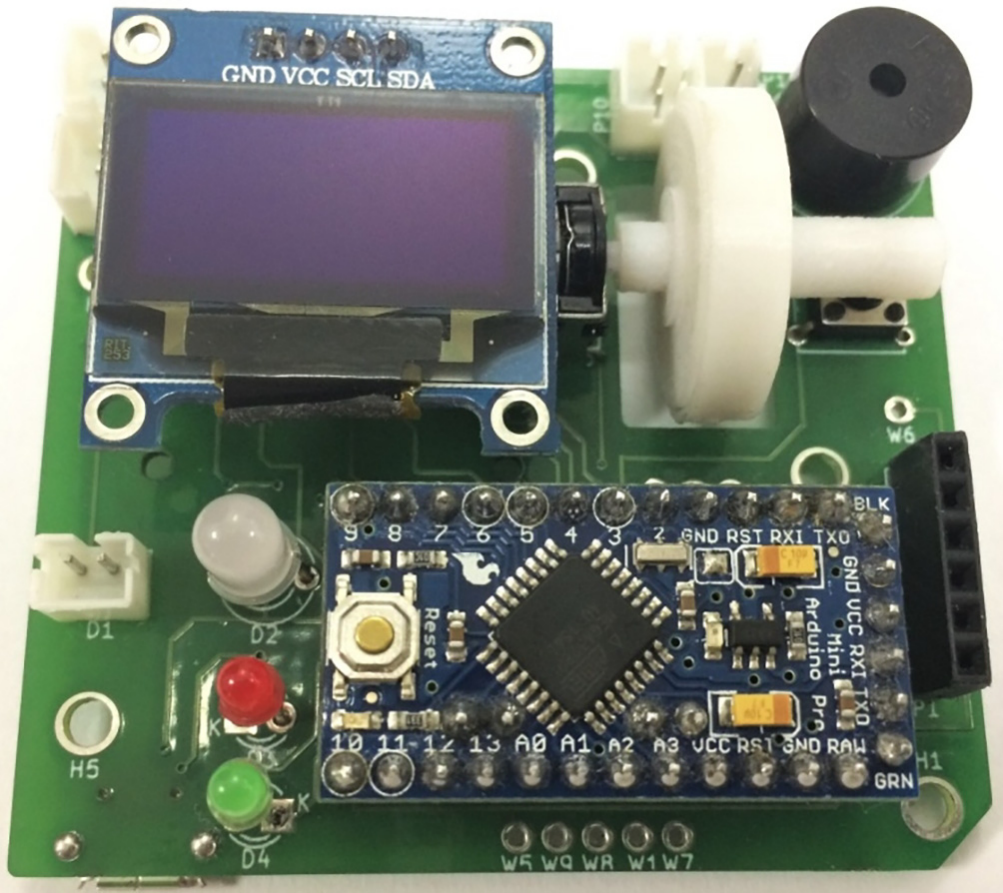
Assembled PCB.

The integrated system with all the modules put together on a single platform is as shown in the Fig. 4. Ki CAD EDA Suite is used for designing the circuit schematic and the layout of the PCB. The hardware assembly consists of a custom Printed Circuit Board (PCB), connectors and other module components.

Once the PCB is fabricated, the Surface Mounted Device components are soldered first, followed by PCB connectors and the other through hole components. Finally, the detachable components are installed onto the PCB connectors along with the scroll wheel. See Fig. 6 for details of assembly steps.

**Fig. 5:**
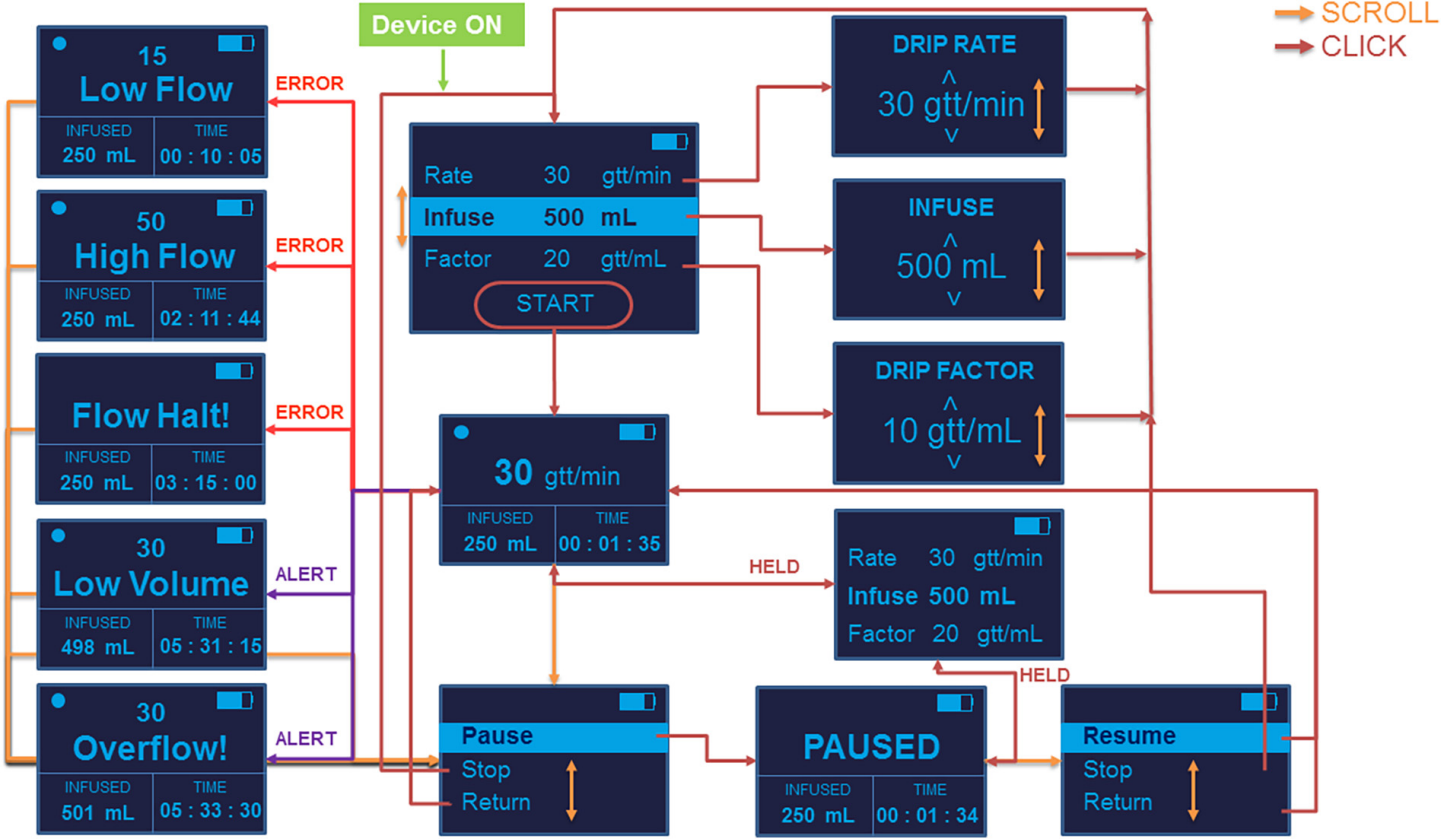
Wireframe of User Interface.

**Fig. 6:**
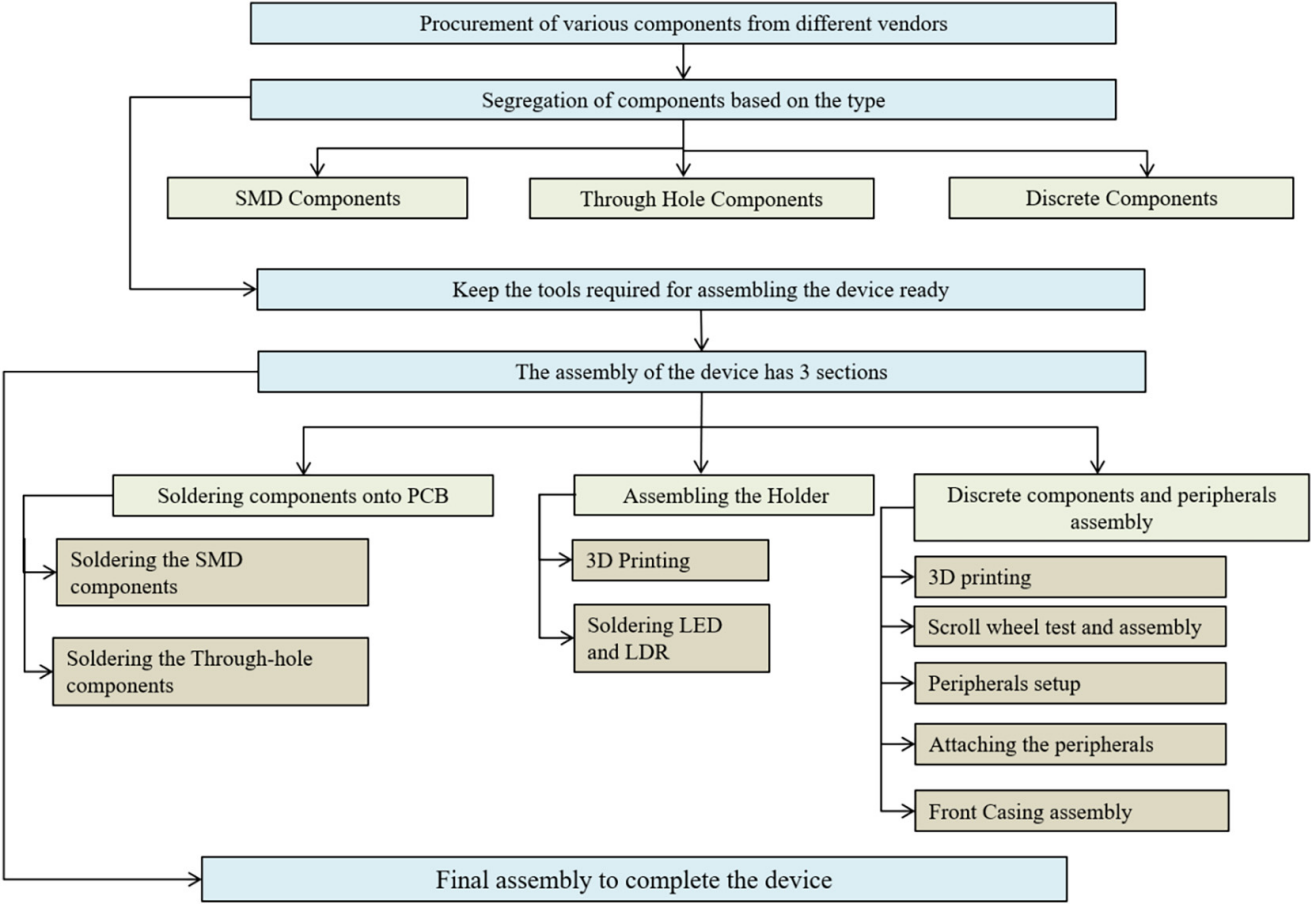
Summary of assembly process.

### Firmware and wireframes

**Fig. 7:**
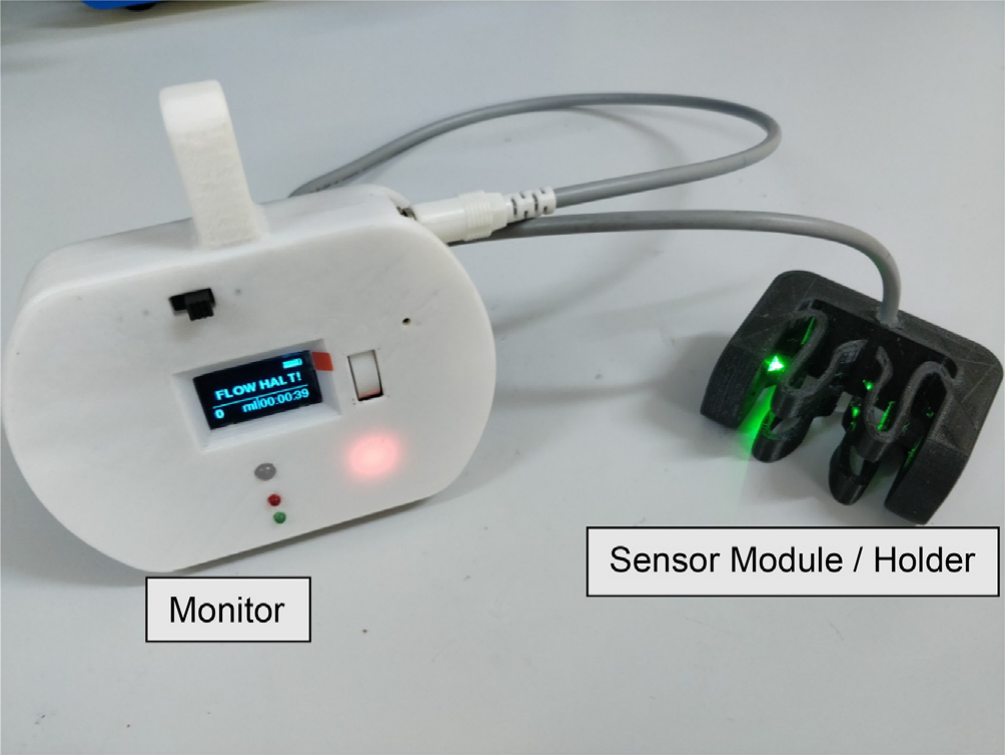
Assembled DripOMeter device.

Firmware for DripOMeter is written in embedded C in Arduino IDE to enable user interactions and processing of drop detector signals and generation of alarms and alerts. Fig. 5 shows the wireframe of the user interface of the system. It also includes the control flow based on the user choice in the form of scroll wheel or tactile switch press. Assembled DripOMeter is shown in Fig. 7.

### Advantages over pre-existing method

#### Novel drop detector

As explained in the section 2.3.1, DripOMeter features a novel drop detection circuit which is designed to accurately detect drops, free from false errors. The drop detector has multiple stages involving voltage divider, a low pass filter to overcome drop debounce and a comparator to generate interrupt whenever a drop is sensed. Compared to the existing systems, drop detector adds extra safety to the entire system.

#### Multiple session alerts

DripOMeter offers thorough monitoring of the infusion sessions along with multiple session alerts. It provides high and low infusion alerts for drip rate deviation, flow halt alert, low volume alert and over-infusion alert. This adds an extra layer of safety for the system, compared to the pre-existing methods which featured only a couple of session alerts.

#### Multiple parameter options

DripOMeter provides options to set drip factor, volume to be infused and drip rate values which makes it usable with various drip set types and different types of fluid infusions. Also, DripOMeter stores the set parameters and automatically pre-sets the same for the next session.

### Functionalities of the hardware

DripOMeter basically monitors the various parameters of an IV drip session [16]. The functionalities of the system can be listed as follows-.

• **Drip rate estimation**: Different drip rates are set for infusing different types of fluids. One of the primary functions of this system is to calculate the drip rate set by the user during the session by counting the number of drops falling into the drip chamber and considering the time gap between two successive drops. Drip rate is expressed in gtt (drops)/minute.
• **Infusion volume estimation**: Volume to be infused is an important session parameter as it determines the amount of fluid that enters the subject’s body. The system is designed to measure the volume of the fluid infused based on the number of drops detected and considering the drip factor of the drip chamber in use. Infusion volume is expressed in millilitres.
• **Session time estimation**: Session time is the overall time for which the infusion process is carried out. Once the user sets the session parameters and begins the session, the system starts the session timer using the internal hardware timer. The session time is displayed on the session screen in the hour: minute: second format.
• **Session error alerts**: The IV drip sessions are subjected to multiple errors that can occur due to various reasons during the course of the session. Changes in drip rate can happen due to various factors like change in height of the reservoir, changes in venous pressure of the patient’s vein, decrease in pressure due to reduction in the reservoir volume, occlusion of the system etc., because of which less or more than intended fluid is administered into the patient. The system is capable of detecting the errors and generates the alerts to draw the attention. Following are the alert features included-
  o **Drip rate deviation alerts**: To detect the drip rate deviation from the set drip rate, a deviation factor is pre-set. The difference between the estimated drip rate and the user-set drip rate is calculated and compared with the deviation factor. Based on this comparison, the higher flow and lower flow conditions of the drip flow rate are identified, resulting in display and sound alert.
  o **Drip halt alert**: The halt in the drip flow can occur due to a blood clot or due to the deformation in the IV drip tube. The system continuously monitors for the session errors and if, a drop doesn’t occur within a pre-set halt time, then it is considered as drip halt error and the user is alerted.
  o **Infusion volume alerts**: The system is designed to check whether the infused volume is near to the set infusion volume parameter and generates an alert, if the difference between the infused and set infusion volumes becomes equal to the pre-set low volume factor. Also, an overflow alert is provided when the infused volume exceeds the set infusion volume.

## Design files summary

Table 1 provide summary of design files associated with DripOMeter.

• *DripOMeter Hardware* is the repository containing schematic, PCB layout and gerber files of the DripOMeter designed using Ki CAD software
• *DripOMeter Software and Firmware* is the repository that has the main program and the flowchart of the DripOMeter. The main program is in Embedded C format using Arduino IDE, which is to be uploaded to Arduino Pro Mini. The main program is responsible for bringing about all the functionalities of the system to reality.
• *DripOMeter CAD* is the repository containing Solid Works and STL files for various parts of DripOMeter.

**Table 1:** Design files summary.

**Design file name**	**File type**	**Open-source license**	**Location of the file**
DripOMeter Electronic Hardware	Ki Cad Project	CERN Open Hardware License Version 2 -Weakly Reciprocal (CERN-OHL-W)	https://doi.org/10.17605/OSF.IO/DYCMS
DripOMeter Software and Firmware	Arduino sketch (.ino)	CERN Open Hardware License Version 2 - Weakly Reciprocal (CERN-OHL-W)	https://doi.org/10.17605/OSF.IO/DYCMS
DripOMeter CAD	Solid Works and STL Files	CERN Open Hardware License Version 2 -Weakly Reciprocal (CERN-OHL-W)	https://doi.org/10.17605/OSF.IO/DYCMS

## Bill of materials summary

Table 2 provide summary of bill of materials used for fabrication of the DripOMeter. A detailed BOM is available in the repository along with other design files. Link for the same: https://osf.io/bnwex/.

**Table 2:** Bill of materials summary.

**Sl. No.**	**Component**	**Number**	**Total cost ($)**
1	Printed Circuit Boards	1	6.37
2	3D printed parts: Back Casing Front Casing Scroll Wheel LDR Cap LED Cap Holder	1 each	23.41
3	Through -hole components	Refer detailed BOM	16.55
4	SMD components	Refer detailed BOM	5.44
5	Peripherals	Refer detailed BOM	7.45
**Total**			59.22

## Build instructions

The flow chart briefly describes the steps involved in building the DripOMeter device. The assembly of the device has 3 main sections:

1. Assembling the Monitoring device
2. Assembling the Holder
3. Discrete components and peripherals assembly and interfacing the Monitor and Holder

.

The detailed instructions for assembling the devices are provided in the document called DripOMeter Build Instructions which is uploaded to the repository.

## Operation instructions

For safe and proper operation of the DripOMeter, the following instructions need to be followed-.

i. Care must be taken that the drip set is attached properly to the reservoir such that, the drip chamber axis is in match with the reservoir axis. Also, it is recommended for the drip set to be held on an IV stand.
ii. The drip factor of the drip chamber must be known and confirmed.
iii. DripOMeter must be properly placed on the drip chamber such that, the drop falling into the chamber intersects the light beam formed inside the drop detector.
iv. The drip chamber should not be subjected to shaking, which can lead to the drop going undetected by the DripOMeter.
v. At the beginning of the infusion session, care should be taken that the battery is fully charged, so as to monitor the entire session uninterrupted. Though the DripOMeter can be charged while in use, it is not advisable to do so as it reduces the mobility of the IV stand.
vi. Users must make sure that the session parameters are set properly. Also, the set parameters are stored in memory for the future sessions.
vii. Users can start the drip flow and set the drip rate after selecting the start session option in the DripOMeter. Drip rate is displayed on the screen and the alarms are disabled for a minute to assist the user to adjust the drip rate.
viii. To pause the session, users can select the pause option by scrolling through the menu and halting the drip flow. To resume the session, users must select the resume option and adjusting the drip rate.
ix. Once the session begins, users can check the set parameters by pressing and holding the scroll wheel.
x. On occurrence of an error, alert is provided to the users in the form of display notification and the audible alarm. Users can silence the alarm for some time by pressing down on the scroll wheel.
xi. Once the users select the stop option, the session details will be lost.
xii. For better battery health, the overcharging of the battery should be avoided by discontinuing the charge session as soon as both the green and red small LEDs are lit up.
xiii. From time to time, the system can be calibrated by comparing the infused volume estimated by the system with the measured volume using graduated cylinder or electronic weighing scale.

## Validation and characterization

### Validation

**Fig. 8:**
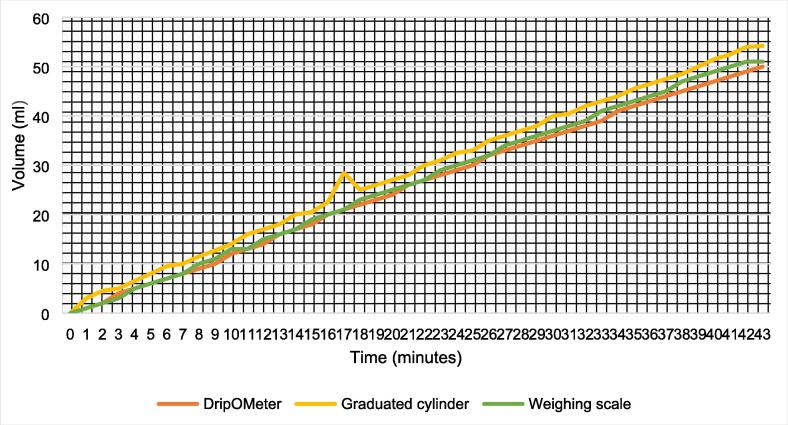
DripOMeter validation.

For the validation of the DripOMeter, a graduated cylinder and calibrated electronic weighing scale were used as standard devices. In this process, the drip being infused was collected in the graduated cylinder which was placed on an electronic weighing scale. Volume infused during a session as estimated by the DripOMeter is compared to the volume collected by the graduated cylinder and the volume equivalent of the weight measured by the weighing scale. Fig. 8 shows the graphs of the test observations. Following parameters were set for the validation session.

Drip Rate = 30 gtt/minute.

Total Volume to be infused = 50 ml.

Drip Factor = 20 gtt/mL.

Weight of the empty cylinder and the tube section present in it = 105 g.

From the above test observations, it is evident that the volume calculated using weight measurement and the reading of graduated cylinder is in close match with the DripOMeter estimated volume, offering the following conclusions:

i. Drip factor set for the drip chamber used is correct.
ii. DripOMeter does not miss the drops passing through the drip chamber.
iii. Total infusion volume estimation performed by the DripOMeter is correct.

Thus, successfully validating the DripOMeter against the standard graduated flask and the electronic weighing scale.

### General testing

**Fig. 9:**
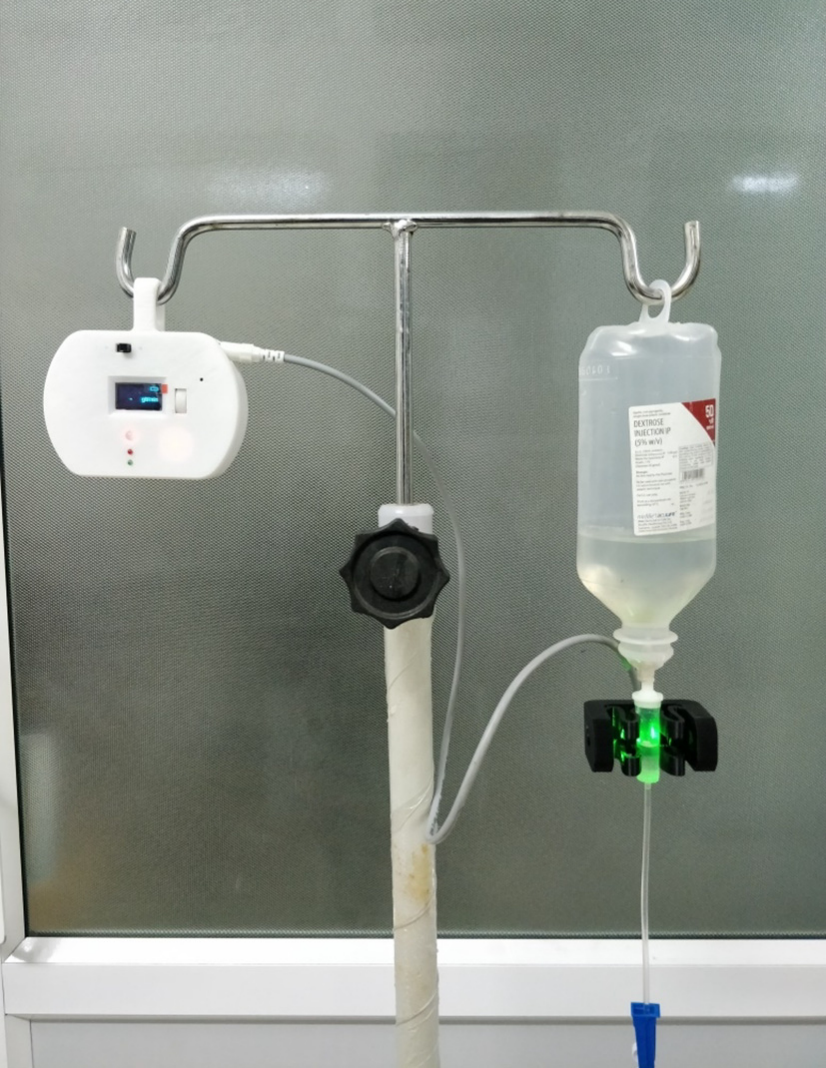
System in use.

DripOMeter hardware was subjected to various tests to ensure its performance during use conditions (Fig. 9). They are as follows:

#### Diagnostic test

Diagnostic test is the self-test performed by the system. It is in the form of the Arduino program for testing DripOMeter PCB with the assembled components. The test checks for the following:

i. Proper track connections in PCB
ii. Working status of the components
iii. User Interface

DripOMeter successfully completed all the tests that were a part of diagnostic test thus, indicating that the assembled PCB functions properly.

#### Load test

As the sensor module of the DripOMeter is held on the drip chamber it acts as a load and can potentially dislodge the drip chamber from the drip reservoir. Thus to test robustness of typical connection between drip chamber and drip reservoir the drip chamber was subjected to an additional load with multiple standard weights being applied in the increasing order. It was observed that loads of up to 300 g have no effect on the drip chamber position even for extended duration of time. As the weight is increased above 300 g, the drip chamber begins to slide. As the weight of the sensor module is way lesser (approximately 50 g) than the threshold weight i.e. 300 g the chances for drip chamber slipping out drip reservoir because of DripOMeter use are minimal.

#### Room lighting condition

The drip monitoring system uses opto-electronic technology to detect the drops being administered. The lighting condition of the room where the system is located can interfere with the drop detection system. To overcome this, a mask with a slit has been added in the sensor module which obstructs the unintended room light from reaching the detector. Also, a dedicated comparator circuit with voltage threshold has been tuned to detect the obstruction to the light caused only by the falling drop.

To test robustness of this design, the system has been subjected to tests by placing it in a room with varying levels of no lighting as well as ambient lighting. The system performance was same in both the cases. No drops went undetected even in the presence of ambient light, thus making the system stable to be used in all types of room lighting conditions.

#### Drop detector orientation

The LED-LDR arrangement in the drop detector is such that the drop obstructs the beam of light relayed from LED to LDR, resulting in reduction of light falling on the LDR. For optimal detection, the detector orientation should be such that the drop falls through the light beam all the time. But, in real life scenarios, the drip chamber may be away from the reference position due to the pull on the IV tube attached to the patient. As a result of this falling drops might fail to block the light beam beyond the threshold needed for the detection circuit. The system was subjected to tests wherein the drip chamber position was varied and its tilt was measured form the reference position. The detector continued to detect the drops even when it was tilted up to an angle of 20°; indicating that the partial obstruction created on LDR was sufficient for detection by the DripOMeter circuit.

### Preliminary validation from stakeholders and future design iterations

A preliminary clinical observation and investigation of the device was carried out at various hospitals after seeking the approval of authorities [17]. The observations were conducted under the supervision of medical staff without disturbing the diagnosis and other procedures involved in the treatment.

The first part of the study involved validation of design from the stakeholders regarding various aspects of the design, which include choice of human machine interface, embodiment design and mounting modality of the device. The device was operated by the nursing staff without connecting the IV drip set to the patient, the following design iterations were suggested:

• Mounting modality: Compact unibody embodiment with simpler mounting modality
• Human Machine-Interface design: Inclusion of larger display with conventional button/ keypads for input controls
• Emergence of new functional requirements: Data logging of key parameters, inclusion of visual alerts and provision to change alarm parameters are a few new requirements that were proposed by clinicians or were observed during the operation of the device.

The second part of the study involved installing the DripOMeter to an IV Drip Set to verify the values displayed by the device in comparison to the parameters.

Three trials were conducted without connecting the device to patients to understand the usability of the device by the nursing staff.

• In the first case, the nurse set the rate of fluid to be infused at 100 ml/hr (25 gtt/min). The DripOMeter was installed on the IV drip system, with the drip chamber having the drip factor of 15gtt/ml. Observations were made for 30 min and the rate measured from DripOMeter was in the range of 22–26 gtt/min.
• In the second trial, the nurse set the rate of the fluid infusion to 50 ml/hr (12.5gtt/min). Observation was done for 30 min. The infusion of fluid was stopped for few minutes using the roller clamp. DripOMeter detected the same and the ‘Flow Halt’ alert was displayed in this period.
• In the third trial, the fluid was to be administered at the rate of 180 ml/hr (45 gtt/min). The parameters for the device were set and corresponding alarms enabled. The volume of fluid to be infused was set at 50 ml and the drip rate was set at 45gtt/min. As anticipated, as per the parameters set in the DripOMeter, there was an alarm after 40 ml of volume infusion and on completion of 50 ml of volume infusion. The flow rate detected by the device was also around 45 gtt/min.

Based on these trials, usability and functionality of the DripOMeter is demonstrated by the intended users though not connected to any patient yet. The device also provided proper alarm indications based on the set parameters as per clinical requirements.

The work presented in this paper, describes the design and development of DripOMeter, an open-source optoelectronic system for intravenous infusion. The system is an IV drip monitoring device designed for resource-constrained settings with a low staff to patient ratio. Prototype was tested in laboratory conditions. Preliminary observational investigation at clinical site and interactions with the stakeholders served as a platform to test the usability and functionality of the device in the clinical environment and to obtain feedback on further design iterations. Insights and requirements captured during the preliminary investigation can be collated to further development of the opensource design of the system.

## Ethics statements

Approval for the preliminary investigation was obtained from the Institute human ethics committee (IHEC) of the Indian Institute of Science (IISc), Bengaluru (IHEC approval no: 9/20200821) to undertake the study. Written consent was also obtained from the officer-in-charge of each clinical site.

## Declaration of Competing Interest

The authors declare that they have no known competing financial interests or personal relationships that could have appeared to influence the work reported in this paper.
