# Supplementary material for: DripOMeter: An open-source opto-electronic system for intravenous (IV) infusion monitoring
Source: HardwareX. 2022 Aug 9;12:e00345. doi: 10.1016/j.ohx.2022.e00345 (PMC9418544; doi:10.1016/j.ohx.2022.e00345)
Supplement: Supplementary data 1 [file mmc1.zip › DripOMeter Build Instructions.pdf]

DripOMeter: An Open-source Opto-Electronic System for Intravenous (IV) Infusion Monitoring

Build Instructions

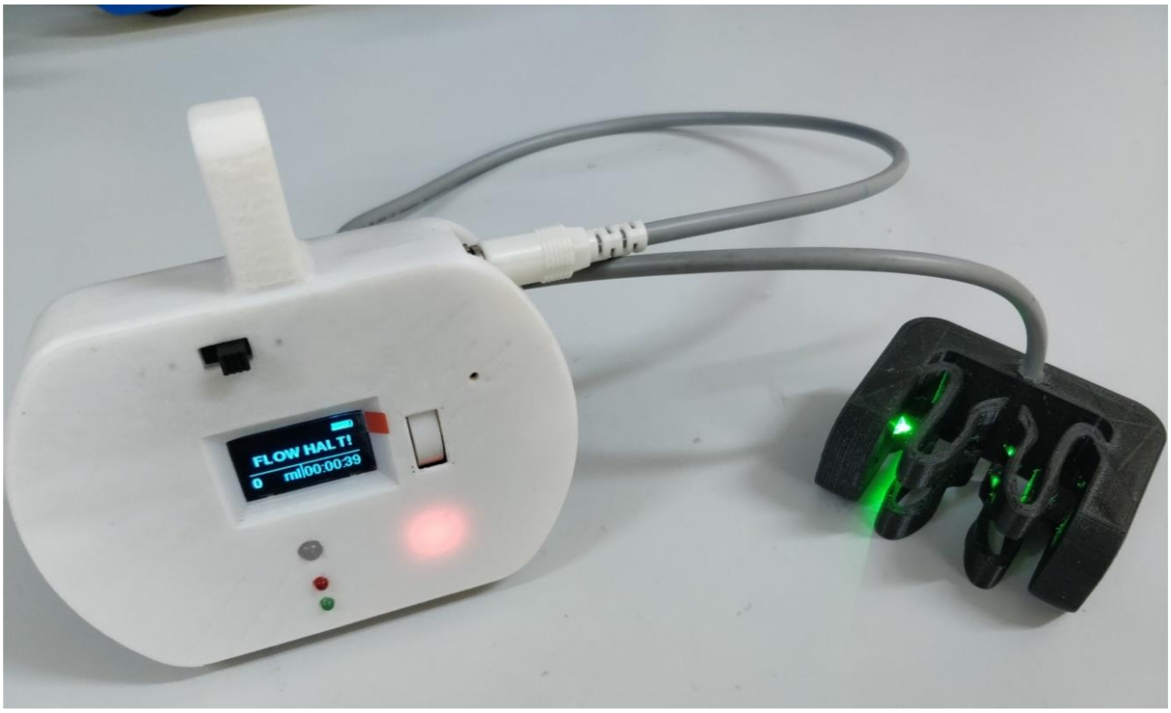

DripOMeter

License: CERN Open Hardware License Version 2 -Weakly Reciprocal (CERN-OHL-W)

Developed at:
UTSAAH Laboratory
PI: Dr. Manish Arora
Centre for Product Design and Manufacturing
Indian Institute of Science, Bangalore

The assembly procedure for the IV Drip Monitoring Device can be categorised based on the following sub-assemblies as shown in Fig. 1.

1. **Stage 1**: PCB Fabrication and assembly
2. **Stage 2:** Holder assembly
3. **Stage 3:** Discrete components and peripheral assembly
    a. Scroll wheel fabrication
    b. Back Casing assembly
    c. Front Casing assembly
4. **Stage 4:** Final assembly

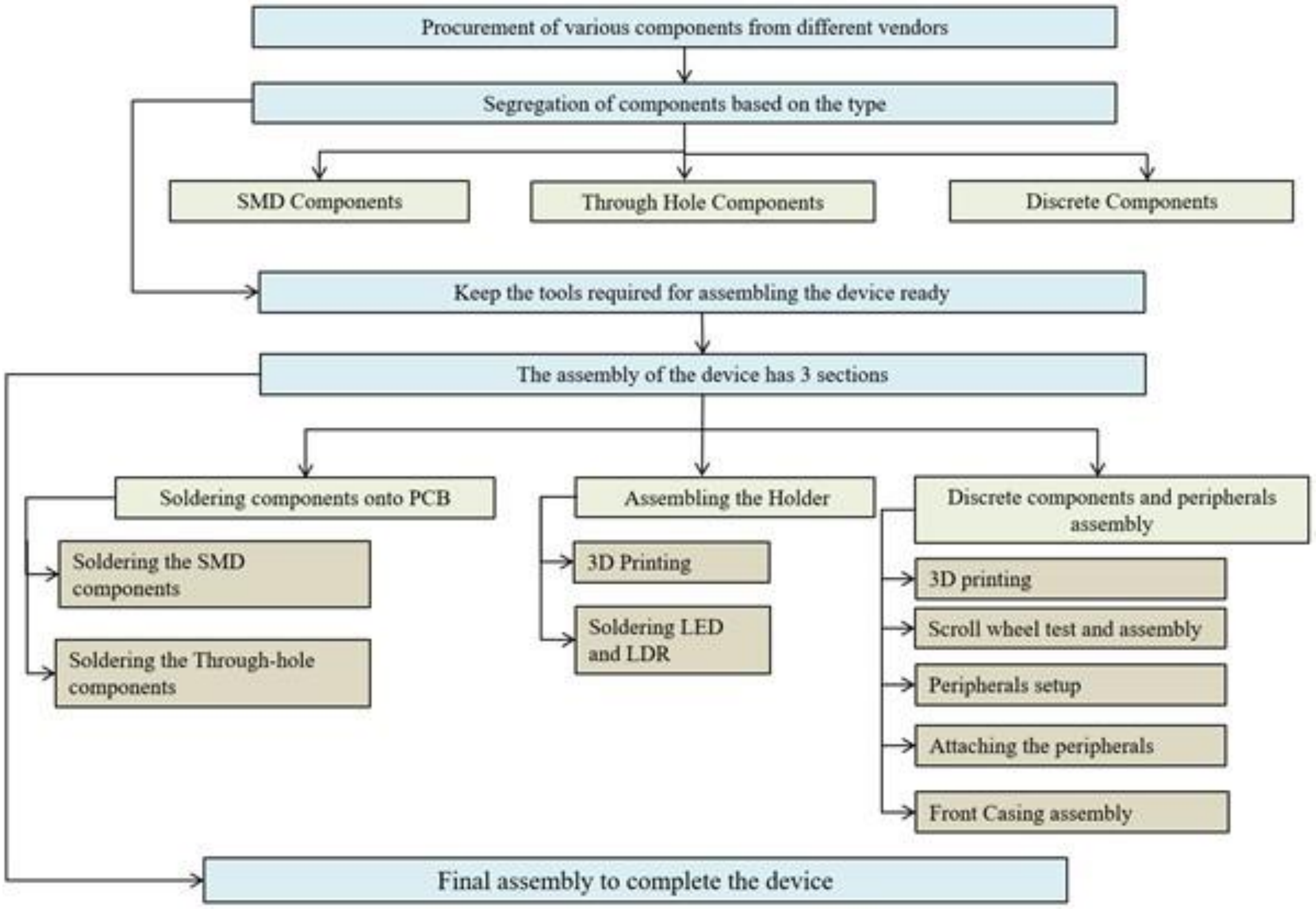

Fig 1: DripOMeter device assembly flowchart

Stage 1: PCB assembly

- Various SMD and through hole electronic components are soldered on to the PCB as shown in Fig. 2. referring to the schematic and PCB layout documents
- The code (provided) in the repository is flashed on to the Arduino Pro Mini using the USB-UART adapter. The Pro Mini is inserted to the respective female headers on the PCB

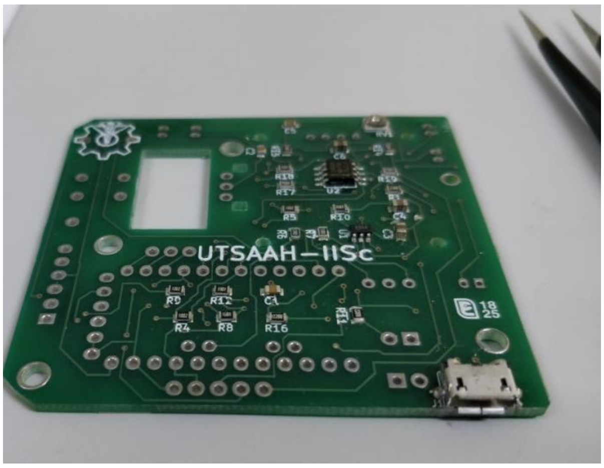

Fig 2: Soldered PCB

Stage 2: Holder assembly

- Holder and the caps are 3D printed
- 4 core cable is inserted through the holder
- LED and LDR are soldered and inserted
- Caps are fitted to the holder
- The outer end of the 4core wire is soldered with the male audio jack

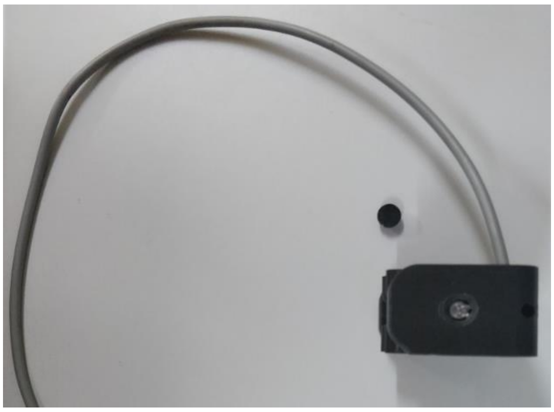

Fig 3: LED and LDR attached to the holder

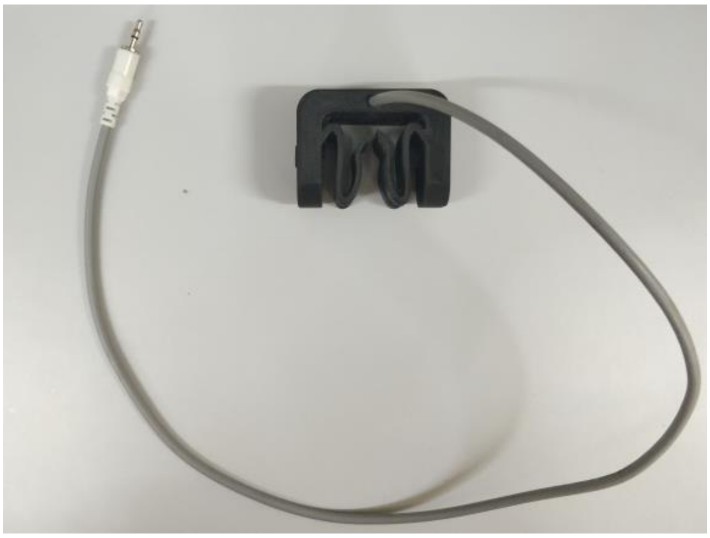

Fig 4: Assembled holder

Stage 3: Discrete components and peripheral assembly

1. **Scroll wheel fabrication**
   - Scroll wheel is 3D printed and assembled to the PCB

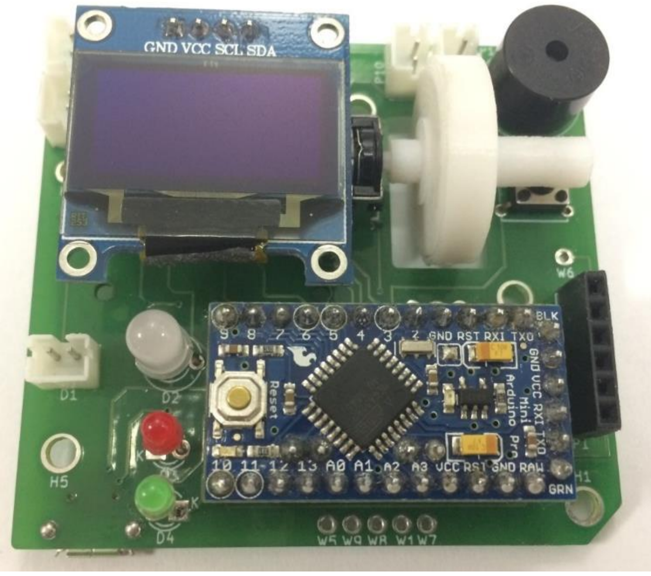

Fig 5: Assembled PCB

2. **Back casing assembly**
   - The back casing is 3D printed
   - Peripherals are attached to the PCB as shown in Fig. 6.
   - PCB with scroll wheel is screwed to the back casing as shown in Fig. 7.
   - Battery clips are attached, and battery is inserted to the back casing

- Female audio jack is connected to the PCB and placed on the back casing

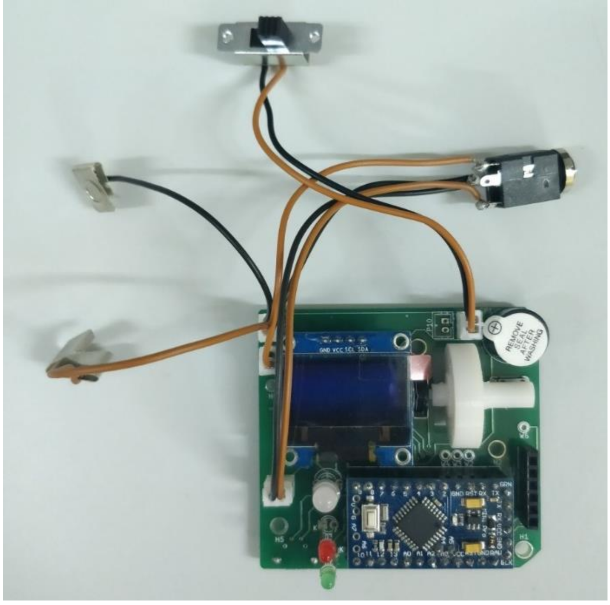

Fig 6: PCB with discrete components

3. Front casing assembly

- The front casing is 3D printed using the corresponding STL file.
- OLED is screwed to the same
- Power switch is attached to the PCB and then screwed to the front casing

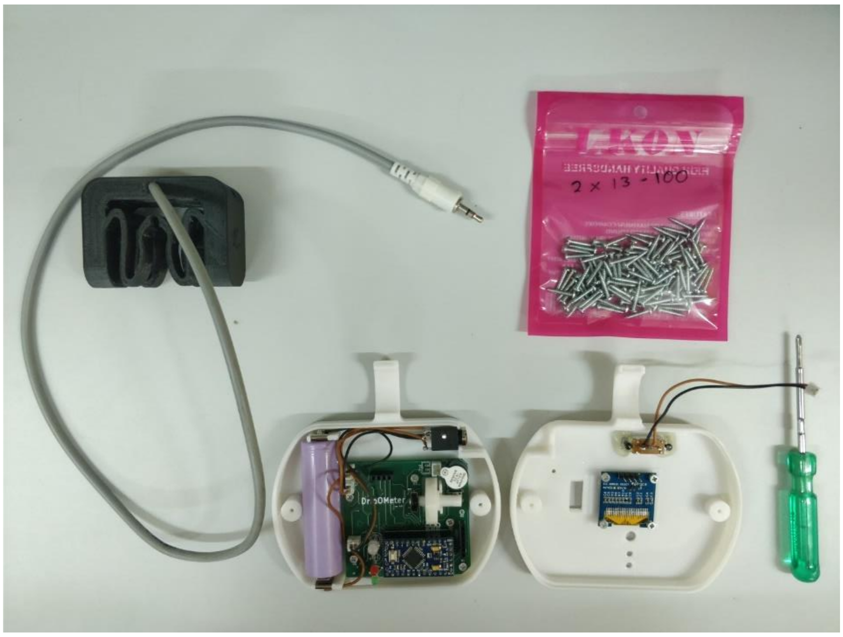

Fig 6: Peripherals assembled to front and back casing

Stage 4: Final Device assembly

- The front casing is snapped to the back casing and fastened
- The scroll wheel action can be tested with the front casing
- Connect the Holder to the 3-contact audio connector
- Switch on the SPDT switch
- Verify the working of all the components and peripherals

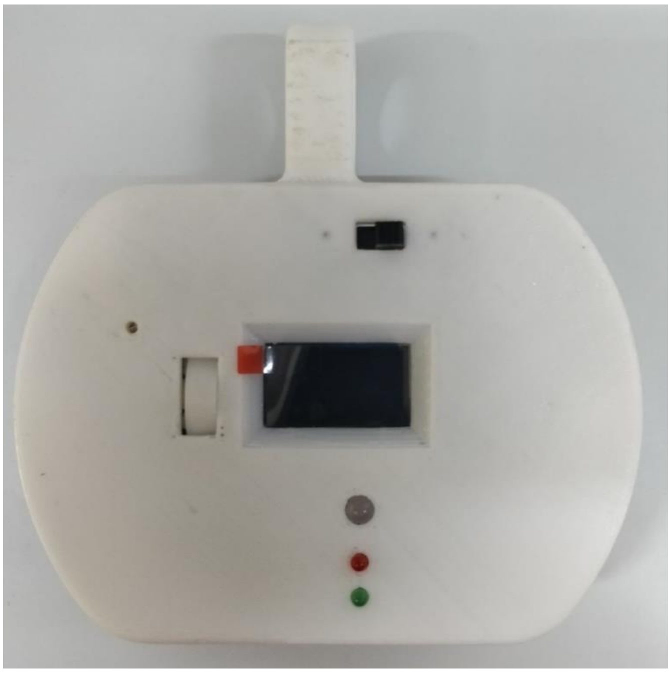

Fig 7: Assembled monitor

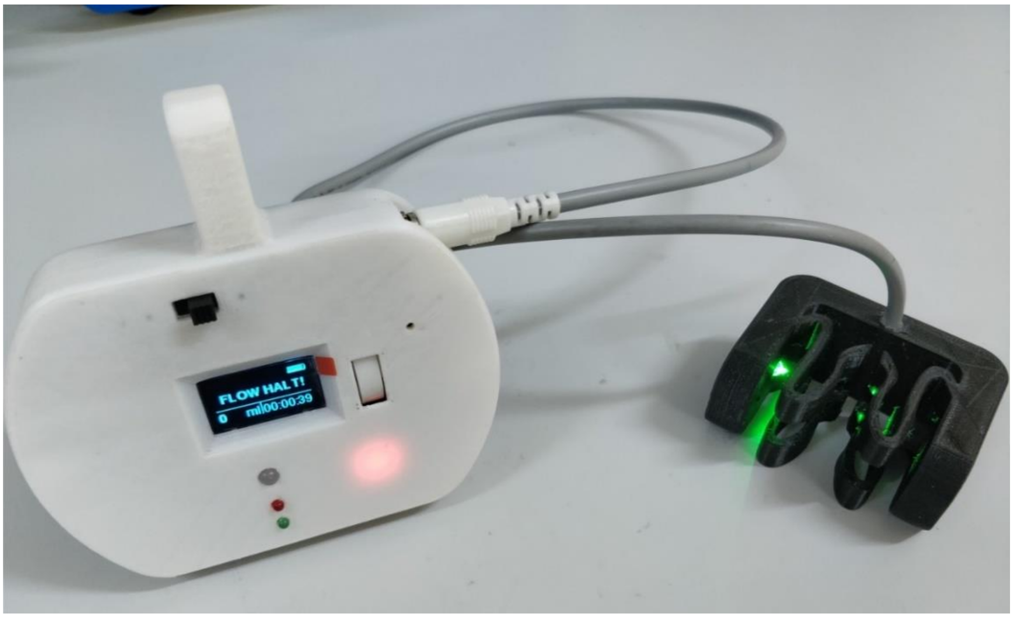

Fig 8: DripOMeter
