# Supplementary material for: DripOMeter: An open-source opto-electronic system for intravenous (IV) infusion monitoring
Source: HardwareX. 2022 Aug 9;12:e00345. doi: 10.1016/j.ohx.2022.e00345 (PMC9418544; doi:10.1016/j.ohx.2022.e00345)
Supplement: Supplementary data 1 [file DripOMeter.pdf › DripOMeter Hardware/DripOMeter.pdf]

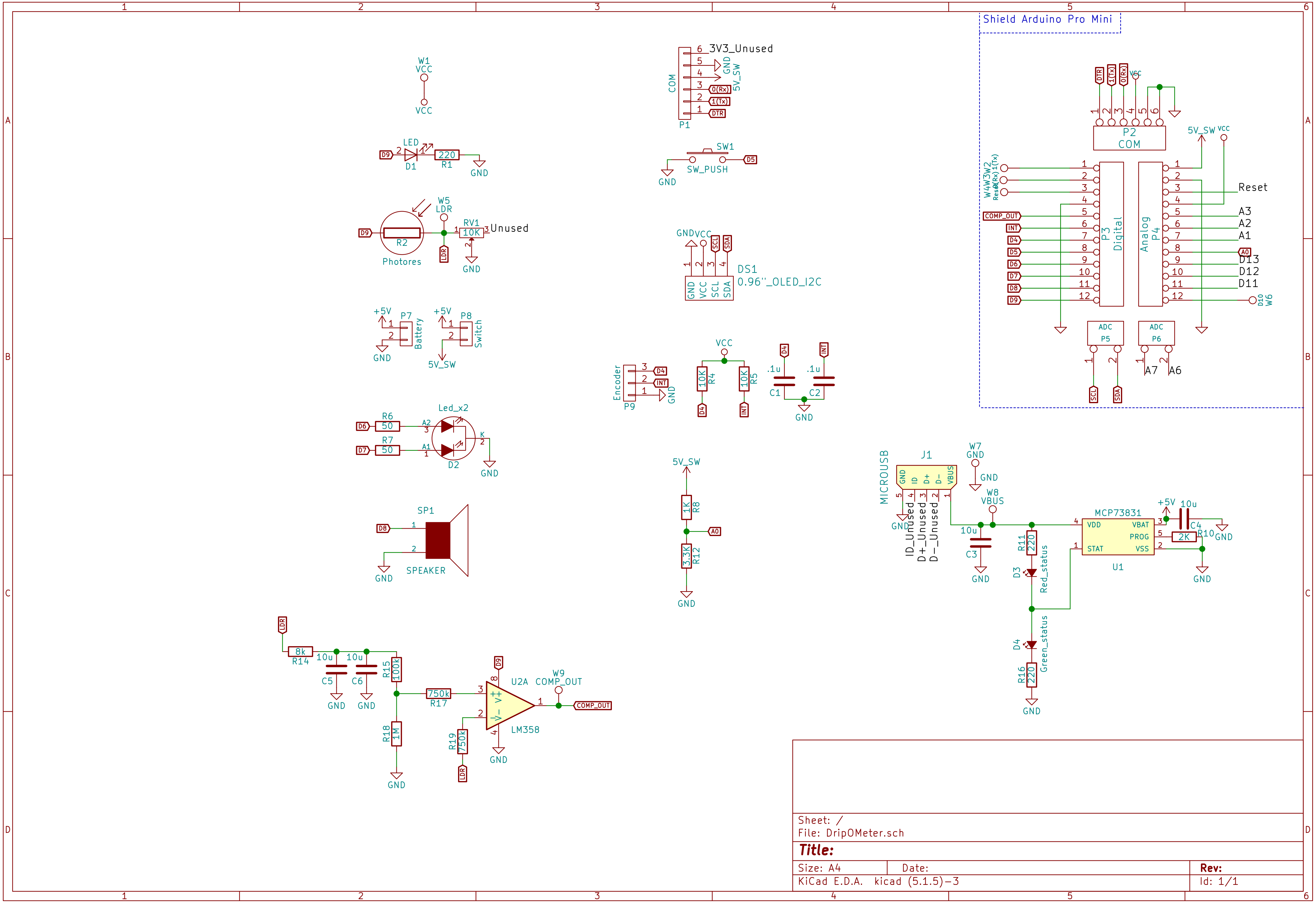

Shield Arduino Pro Mini
W1 VCC
LED D1 R1 220 GND
W5 LDR RV1 10K Unused
R2 Photores
P7 Battery +5V GND
P8 Switch +5V 5V_SW
Led_x2 R6 50 R7 50 D2 GND
SP1 SPEAKER GND
P1 COM 3V3_Unused GND 5V_SW
SW1 SW_PUSH GND
DS1 0.96"_OLED_I2C
Encoder P9
VCC R4 10K R5 10K
C1 .1u C2 .1u GND
5V_SW R8 1K R12 3.3K GND
P2 COM
P3 Digital
P4 Analog
Reset A3 A2 A1 D13 D12 D11
COMP_OUT INT D4 D5 D6 D7 D8 D9
P5 ADC P6 ADC A7 A6
J1 MICROUSB
ID_Unused D+_Unused D-_Unused
W7 GND W8 VBUS
C3 10u
MCP73831 U1
+5V C4 10u R10 2K
R11 220 D3 Red_status
D4 Green_status R16 220
R14 8k C5 10u C6 10u R15 100k
R17 750k R18 1M R19 750k
U2A LM358 W9 COMP_OUT
Sheet: /
File: DripOMeter.sch
Title:
Size: A4
Date:
Rev:
KiCad E.D.A. kicad (5.1.5)-3
Id: 1/1
